# Safety and efficacy of ultrasound-guided thermal ablation in treating T1aN0M0 and T1bN0M0 papillary thyroid carcinoma: A meta-analysis

**DOI:** 10.3389/fendo.2022.952113

**Published:** 2022-07-27

**Authors:** Mei-Huan Wang, Xiao Liu, Qian Wang, Hua-Wei Zhang

**Affiliations:** ^1^ Department of Ultrasound, Shandong Provincial Hospital Affiliated to Shandong First Medical University, Jinan, China; ^2^ Department of Ultrasound, Shandong Provincial Hospital Affiliated to Shandong University, Jinan, China

**Keywords:** papillary thyroid carcinoma, thyroid nodule, thermal ablation, radiofrequency ablation, microwave ablation, laser ablation, meta‐analysis

## Abstract

**Background:**

Papillary thyroid cancer (PTC) is the most common thyroid tumor, and early diagnosis and treatment can effectively improve prognosis. Many controversies surround the treatment method of T1N0M0 PTC. Recently, thermal ablation (TA) has shown some benefits in the treatment of PTC patients, but the safety and efficacy of its treatment remain controversial. This article performs a meta-analysis of TA in patients with T1aN0M0 and T1bN0M0 PTC.

**Methods:**

The PubMed, Embase, Web of Science, and Cochrane Library databases were systematically searched for retrospective or prospective studies of TA for treating patients with T1N0M0 PTC from the database establishment to May 1, 2022. Data on volume reduction rate (VRR), disease progress, and complication rate were collected. In addition, a meta-analysis was performed using the Stata 12.0 and Review Manager 5.3.

**Results:**

A total of 9 eligible studies were included. Our study demonstrated the effectiveness of VRR and disease progress. The VRR was reduced after 3 months (−75.90%; 95% CI [−118.46–33.34%]), 6 months (34.33%; 95% CI [15.01–53.65%]), 12 months (78.69%; 95% CI [71.69–85.68%]), and 24 months (89.97%; 95% CI [84.00–95.94%]). The disease progress was 1.9% (95% CI [1.1–3.0]). Safety is justified by the complication rate, which was 6.5% (95% CI [3.5–10.2]). Pain and hoarseness were the most common complications, and no life-threatening complications were reported. Egger’s test demonstrated that publication bias was acceptable.

**Conclusions:**

TA is an effective and safe method for managing T1aN0M0 and T1bN0M0 papillary thyroid nodules.

## Introduction

Thyroid cancer currently ranks fifth among cancers affecting women worldwide, accounting for almost 5% of the whole cancer burden in female patients (1). Papillary thyroid carcinoma (PTC) is the most frequent type of thyroid cancer, accounting for up to 90% of all thyroid malignancies (2). In spite of the fact that well-differentiated PTC may remain inactive (3), distant metastases and the recurrence rates are approximately 50% and 20%, respectively (4). T1N0M0 PTC is a subgroup of PTC that is characterized by a diameter equal to or less than 2 cm (T1a: ≤1 cm; T1b: >1 cm, ≤2 cm) (5).

Thermal ablation (TA) methods such as radiofrequency ablation (RFA), microwave ablation (MWA), and laser ablation (LA) have recently been deployed for thyroid nodules (6–10). Some studies have reported that TA is a rather effective and safe treatment because of its low incidence of tumor metastasis and complications (11–13). Although TA has been considered a suggested therapy method for T1N0M0 PTC, the results are not reliable enough for the short follow-up duration and small sample size in previous studies. Accordingly, it is necessary to collect and aggregate published data regarding the performance of TA for T1N0M0 PTC and to analyze the efficacy and safety of ablation techniques.

This meta-analysis aims to evaluate the efficacy and safety of TA in treating patients with T1N0M0 PTC by variables such as tumor volume reduction rate (VRR), disease progress, and complication rate and to explore factors causing heterogeneity.

## Methods

### Literature search

We collected our data with the articles published in PubMed, Web of Science, Embase, and Cochrane Library from the establishment of the database to May 1, 2022. The search MESH terms were as follows: [(“papillary thyroid cancer” OR “papillary thyroid carcinoma” OR “papillary thyroid microcarcinoma” OR “thyroid micropapillary carcinoma” OR “papillary thyroid micro-carcinoma”) AND (“radiofrequency ablation” OR “RFA” OR “laser ablation” OR “LA” OR “microwave ablation” OR “MWA” OR “thermal ablation”)]. Article‐related data were further evaluated to identify potential additional eligible data.

### Inclusion criteria

Studies according to all of the following criteria were included: (1) ultrasound-guided biopsy confirmed PTC without distant metastases; 2 cm or less in maximum diameter. (2) patients received only one modality of LA, RFA, or MWA; (3) the experimental methods used in the study were randomized controlled trials, retrospective analysis, or prospective analysis; and (4) the experimental results included VRR, disease progress, and complication rate.

### Exclusion criteria

Exclusion criteria were as follows: (1) patients who have not been treated with ablation; (2) other types of study: review, case reports, letters, and comments; and (3) patient and data overlapping studies.

### Data extraction

The following characteristics were extracted from the included studies: (1) study characteristics: the first author’s family name, publication year, study period, design style, and treatment methods; (2) basic characteristics of the patients: mean age, sample size, sex ratio, and follow‐up interval; and (3) postoperative characteristics of the patients: VRR, disease progress, and postoperative complications. The data were extracted by two researchers independently, and when data are at odds, discussion was conducted by the two researchers.

### Literature quality assessment

Because all the studies included in the analysis here were non-randomized controlled trials, this quality assessment adopted the Newcastle–Ottawa Scale (NOS) (14). Two researchers independently conducted literature quality evaluations using the NOS for cohort study. NOS includes 4 items (4 points) for “Research Subject Selection”, 1 item (2 points) for “Comparability between Groups”, and 3 items (3 points) for “Result Measurement”. The scale is set with a full score of 9 points. When a study ≥7, it is regarded as high-quality literature. Correspondingly, a study with a score of lower than 7 is considered lower-quality literature. When the opinions are inconsistent, it is decided through discussion or consultation with the third person.

### Statistical analysis

Statistical analysis was conducted with Stata 12.0. Continuous variables are presented as the means with standard error (SE). For each continuous variable comparison, the mean difference (MD) was calculated with a 95% confidence interval (CI). *p* < 0.05 is considered to be statistically significant. The *I* ^2^ test evaluates heterogeneity, and *I* ^2^ > 50% indicates significant heterogeneity. When there is significant heterogeneity, the random effect model (DerSimonian–Laird method) is used to calculate the combined effect; otherwise, the fixed model (Mantel–Haenszel method) is used instead. Egger’s test of Stata12.0 version was used to assess possible publication bias.

## Results

### Study characteristics

**Figure 1:**
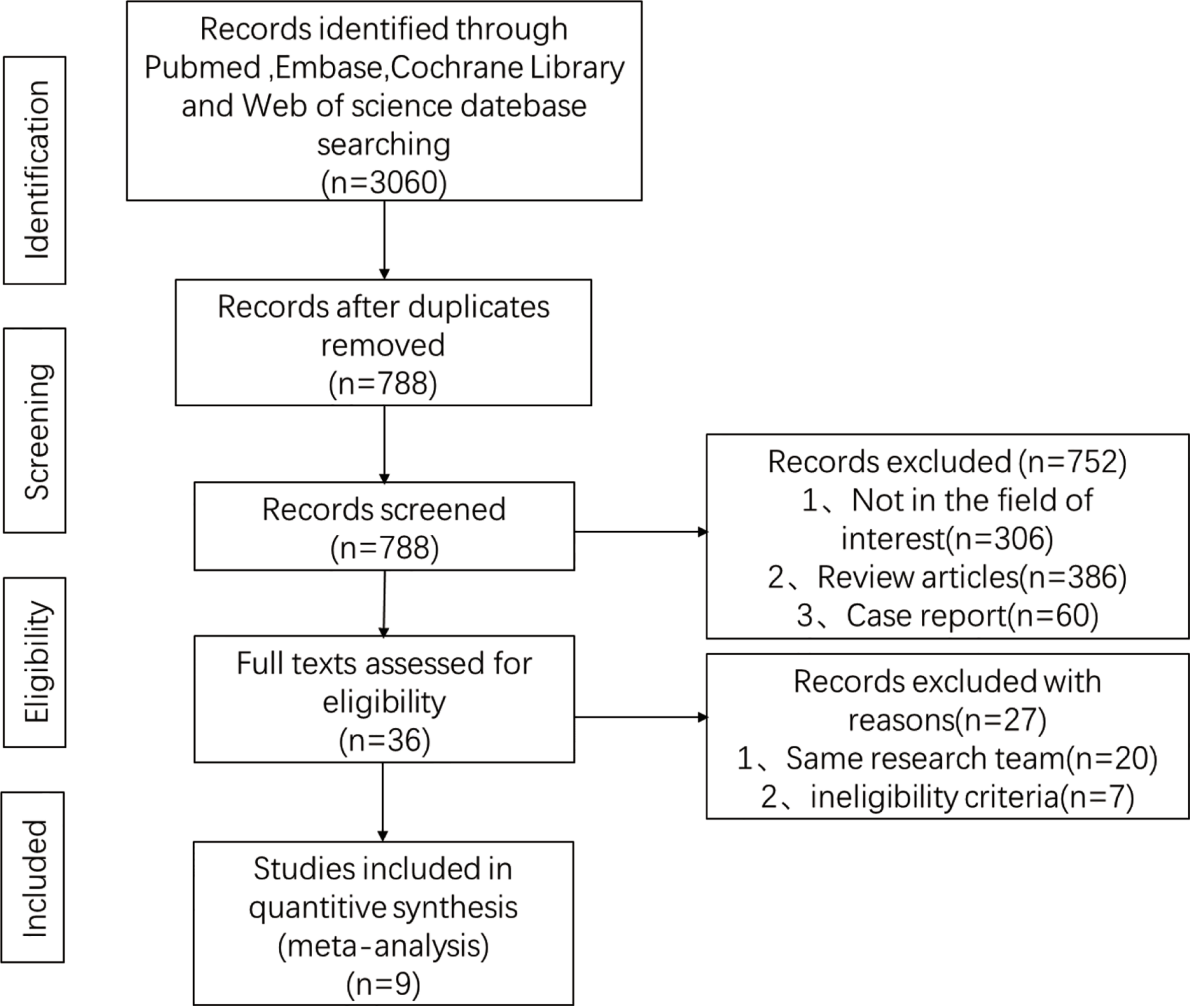
Flow diagram of the study selection process.

The study screening procedure is presented in a PRISMA flowchart (**Figure 1**). A total of 3,060 related articles were collected by searching the databases. After removing 2,272 duplicate articles, 386 review articles, 60 case reports, and 306 unrelated studies, only 36 articles need to be reviewed. To avoid data duplication, only one study of the same set of data from the same team was employed. Finally, 9 articles including 1,215 patients were ultimately enrolled in data synthesis and meta-analysis (15–23). Within the eligible studies, 1 study was prospectively designed while the remaining studies were retrospective. The overall quality of the studies was high based on NOS. All study characteristics are displayed in **Table 1**. In addition, 6 studies collected data on T1aN0M0 PTC, while other ones focused on T1bN0M0 PTC.

**Table 1:** Characteristics of the studies included in this meta-analysis.

Authors	Year	Study Design	Study Period	No. of patients (Female/male)	Age (year) Mean ± SD	Follow-up (months)Mean ± SD	Treatment	Disease Progress	Complication	NOS-Gen
T1aN0M0										
Peng et al. [21]	2021	Retro	2012.6-2015.5	105 (74/31)	44.1±12.2	65.4±6.3	LA	1, 2	pain	8
Teng et al. [23]	2020	Retro	2014.6-2014.10	41 (28/13)	46.10±8.85	NA	MWA	None	hoarseness	7
Wang et al. [22]	2021	Retro	2016.1-2018.12	63 ( 51/12)	43.56± 14.172	NA	MWA	None	reactive lymph node hyperplasia	7
Xiao et al. 17]	2021	Retro	2014.4-2019.12	131 (104/27)	41.2±10.9	26.2±13.3	RFA	1	pain	8
Yue et al. [25]	2020	Pro	NA	119 (92/27)	48.7±11.8	37.2±20.9	MWA	1	hematoma, hoarseness, labored breathing, coughing	7
Zhou et al. (a) [20]	2020	Retro	NA	34 ( 23/11)	41.8 ± 13.4	23.3±4.4	LA	None	hematoma	7
Zhou et al. (b) [20]	2020	Retro	NA	33 ( 26/7)	37.9 ± 10.1	22.8±4.1	MWA	None	hematoma	7
Zu et al. [24]	2021	Retro	2013.7-2020.6	320 (237/83)	44.99 ± 10.62	29.69±17.73	MWA	1, 2	hematoma, hoarseness	8
T1bN0M0										
Cao et al. [19]	2020	Retro	2015.4-2020.3	172 (134/38)	46±13	24.9±14.1	RFA	1,2	hoarseness, fever	8
Xiao et al. [18]	2020	Retro	2014.4-2018.10	66 (52/14)	41.0±9.2	20.5±7.4	RFA	1,2	pain	8
Xiao et al.(b)[17]	2021	Retro	2014.4-2019.12	131 (103/28)	41.1±10.3	25.1±10.6	RFA	1	pain, hoarseness	8

Retro, retrospective; Pro, prospective; SD, standard deviation; NOS-Gen, Newcastle–Ottawa scale scores; NA, data unavailable; Disease Progress:1, new tumor/tumor recurrence 2, Lymph node metastasis.

### Volume reduction rate

**Figure 2:**
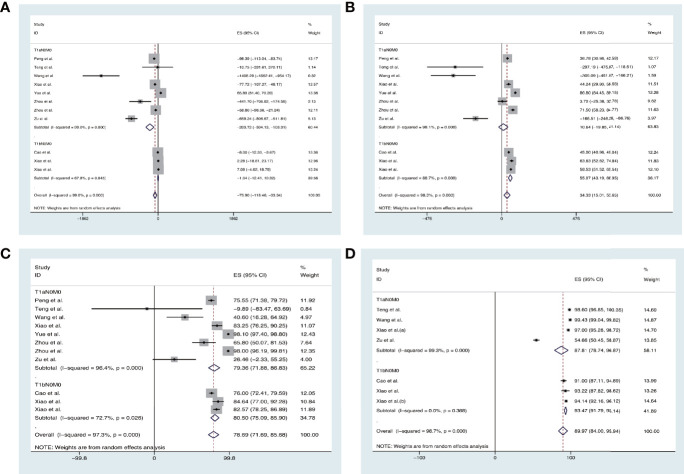
Forest plot of the effect of thermal ablations subgrouped by T1aN0M0 and T1bN0M0 on VAA **(A)** 3 months, **(B)** 6 months, **(C)** 12 months, and **(D)** 24 months after intervention.

The overall pooled estimates for the mean difference (MD) of VRR (the unit is mm^3^) at 3 months, 6 months, 12 months, and 24 months after TA are −75.90% (95% CI, −118.46 to −33.34; *p* = 0.000; **Figure 2A**), 34.33% (95% CI, 15.01–53.65; *p* = 0.000; **Figure 2B**), 78.69% (95% CI, 71.69–85.68; *p* = 0.000; **Figure 2C**), and 89.97% (95% CI, 84.00–95.94; *p* < 0.00001; **Figure 2D**), respectively.

**Figure 3:**
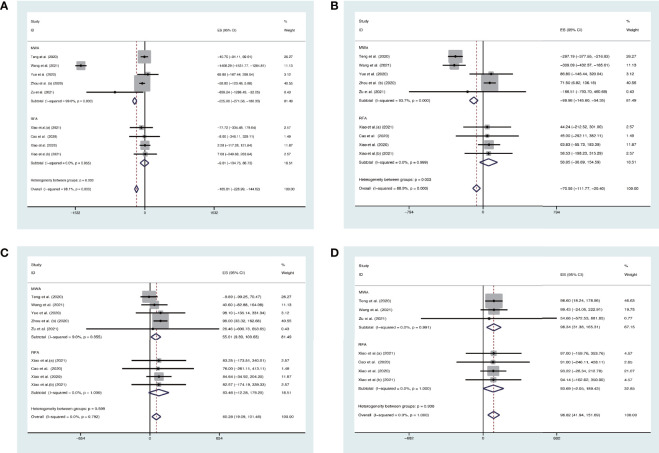
Forest plot of the effect of thermal ablations subgrouped by MWA and RFA on VAA **(A)** 3 months, **(B)** 6 months, **(C)** 12 months, and **(D)** 24 months after intervention.

We also conducted the analysis subgrouped by the different ablation methods. The pooled estimates for the mean difference (MD) of VRR (the unit is mm^3^) at 3 months, 6 months, 12 months, and 24 months after MWA were −225.96% (95% CI, −271.58 to −180.33), −99.98% (95% CI, −145.60–54.35), 55.01% (95% CI, 9.39–100.63), and 98.34% (95% CI, 31.38–165.31), respectively. The pooled estimates for MD of VRR at 3 months, 6 months, 12 months, and 24 months after RFA were −9.01% (95% CI, −104.75–86.73), 58.85% (95% CI, −36.89–154.59), 83.46% (95% CI, −12.28–179.20), and 93.69% (95% CI, −2.05–189.43), respectively (**Figure 3**).

### Disease progress

**Figure 4:**
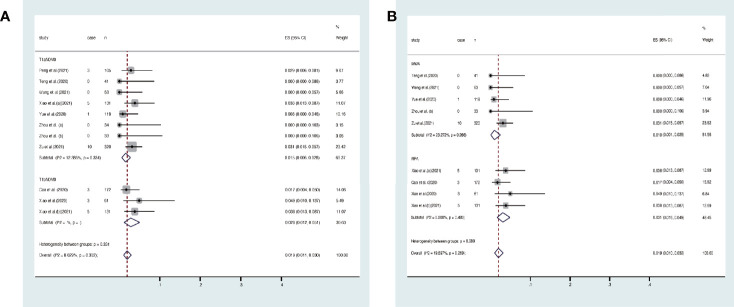
Forest plot of the overall disease progress rate after thermal ablations: **(A)** T1aN0M0 and T1bN0M0 studies, **(B)** MWA and RFA studies.

**Figure 5:**
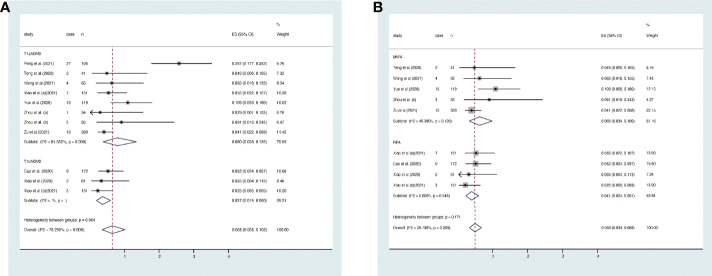
Egger’s funnel plot of the overall disease progress rate: **(A)** T1aN0M0 and T1bN0M0 studies, **(B)** MWA and RFA studies.

Disease progress covered in these articles includes new tumor, tumor recurrence, and lymph node metastasis. At the end of the follow-up, the overall disease progress rate was 1.9% (95% CI, 1.1–3.0%, **Figure 4A**). Specifically, it was 1.5% (95% CI, 0.6–2.8) in T1aN0M0 PTC and 2.8% (95% CI, 1.2–5.1%) in T1bN0M0 PTC. At the end of follow-up, the disease progress rate was 1.0% (95% CI, 0.1–2.5%) in MWA and 3.1% (95% CI, 1.6–4.9%) in RFA (**Figure 4B**). Egger’s test confirmed that the result may have publication offset in both T1N0M0 studies in the analysis (*p* < 0.05, **Figure 5A**) and the studies that were involved in MWA and RFA as the ablation methods (*p* < 0.05, **Figure 5B**).

### Complication rate

**Figure 6:**
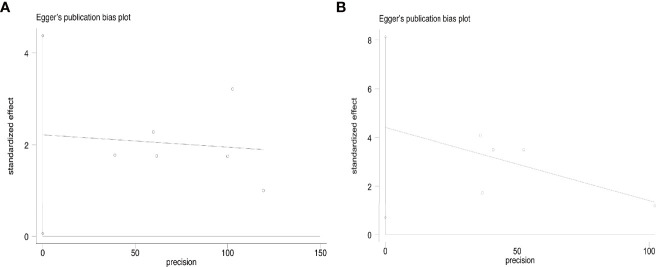
Forest plot of complication rate after thermal ablations: **(A)** T1aN0M0 and T1bN0M0 studies, **(B)** MWA and RFA studies.

**Figure 7:**
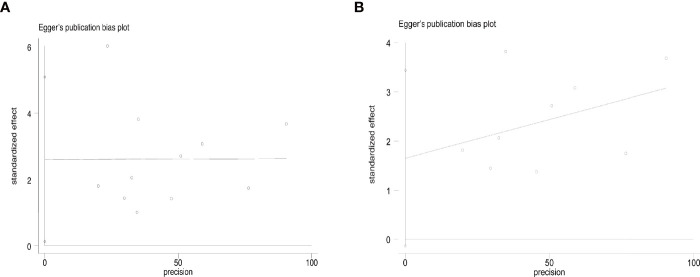
Egger’s funnel plot of the complication rate: **(A)** T1aN0M0 and T1bN0M0 studies, **(B)** MWA and RFA studies.

The overall complication rate was 6% (95% CI, 4–9%, **Figure 6A**). Specifically, it was 8% (95% CI, 4–12%) in T1aN0M0 PTC and 3% (95% CI, 1–5%) in T1bN0M0 PTC. The result of Egger’s test showed an acceptable publication bias (*p* > 0.05, **Figure 7**) in the included studies of T1aN0M0 and T1aN0M0 PTC. When we subgrouped the studies with different ablation methods, the complication rate was 6.3% (95% CI, 3.4–10.0%) in MWA and 4.1% (95% CI, 2.4–6.1%) in RFA (**Figure 6B**). It is confirmed by Egger’s test (**Figure 7**) that the result may have publication offset in the studies of MWA and RFA (*p* < 0.05).

## Discussion

This meta-analysis revealed that US-guided TA is a valid therapeutic method for T1N0M0 PTC. At 24 months after ablation, we observed that VRR was 87.81% in T1aN0M0 PTC and 93.47% in T1bN0M0 PTC. The VRR of T1bN0M0 PTC was preferable than that of T1aN0M0 PTC at 24 months of follow-up, probably because the initial volume of the nodules in T1bN0M0 was greater than that of T1aN0M0, so the relative VRR after TA treatment was more considerable. Though the tumor VRR was not 100% in both subgroups, this may be caused by the following two reasons. First, the absorption of residual tumors by the body is an immune process that takes a considerable long time. The final follow-up period selected for our study was 24 months after TA, which may not be sufficient for adequate absorption of some tumors. Teng et al. (21) showed that the volume significantly decreased from a median of 55.78 (quartile: 21.50, 112.20) mm^3^ to 0 (0, 0) mm^3^ (*p* < 0.001), with a VRR of 99.37 ± 4.02% at 60 months after TA. Second, residual calcification and fibrous scarring within the tumor may be another reason why the tumor volume does not completely disappear. These residues appear as no enhancement zones on contrast-enhanced ultrasound and necrotic material confirmed by fine-needle aspiration biopsies (18).

In the eligible studies, disease progress includes new discovered tumor, tumor recurrence, and lymph node metastasis. No significant differences were shown by disease progress rate between T1aN0M0 [1.5% (95% CI, 0.6–2.8%)] and T1bN0M0 [2.8% (95% CI, 1.2–5.1%)]. For newly observed or recurrent tumors, all patients received secondary TA, while patients with lymph node metastases mostly undergo open surgery. Xiao et al. (16) reported that ablation was performed in a patient with lymph node metastases at ipsilateral level IV, which was successfully treated. TA was shown in our eligible studies to be an effective treatment for T1N0M0 PTC for all cases of tumors and metastatic lymph nodes being cured with no recurrence.

In terms of safety, the main complications were pain (38 patients) and hoarseness (20 patients), with other complications: hematomas (4 patients), slight fever (1 patient), labored breathing, coughing (4 patients), and reactive hyperplastic lymphadenectasis (4 patients). The complication rate in T1aN0M0 PTC and T1bN0M0 PTC was 8.0% and 3.7%, respectively. Although the complication rate was lower in T1bN0M0 PTC, this may be due to the error caused by the smaller sample size in T1bN0M0 PTC, so we do not consider the difference between T1aN0M0 PTC and T1bN0M0 PTC to be statistically significant. Most symptoms were relieved within several months without any specific therapy. Only one person with T1aN0M0 PTC had a permanent recurrent laryngeal nerve injury, showing mild hoarseness without dysphagia and coughing after drinking water (22). In addition, in this meta-analysis, no life-threatening complications were identified; thus, TA is a safe as well as a suitable regimen for treating T1N0M0 PTC.

In our practice, we applied T1bN0M0 PTC, which was not reported in previous studies. Based on our results, TA could be considered as a very promising method to treat T1N0M0 PTC and improve patient outcomes. Compared to surgery, patients treated with TA had shorter operation times and cost less (24). The main reason was that the ablation can be performed in the outpatient clinic through local injection of lidocaine and the procedures are completed rapidly without hospital bills. In terms of complications, those caused by surgery should not be ignored. The incidence of hypothyroidism, as reported, is as high as 75%, whereas that of permanent hypocalcemia is up to 3.1% (25, 26). During the execution of TA therapy, isolation fluid can be injected around the thyroid nodules to prevent thermal injury to important anatomy such as peripheral blood vessels and nerves (27, 28). This also ensures the effectiveness and safety of the TA treatment. In addition, TA is a flexible and repeatable procedure; it can be performed many times for recurrent tumors or metastatic lymph nodes without increased technical difficulties due to the previous treatments (29). This is undoubtedly a gratifying gospel for patients with multiple recurrences of thyroid and lymph nodes. Although good results were seen in our study, the heterogenicity of the results cannot be ignored. Some studies have not been followed up long enough to observe outcomes, which may have affected the accuracy of these results. For example, 3 studies did not follow up for 24 months, which could lead to some tumor recurrences or new tumors not being detected, affecting the accuracy of disease progression. In addition, the data from the same research team may overlap (15, 17), which will also have an impact on the accuracy of our findings.

According to the results of subgroup analysis in the MWA and RFA groups, we discovered that the “tumor volume” of the MWA group was greater than that of the RFA group in the short term after treatment, but after a long-term follow-up, both subgroups showed good VRR, and the difference was not statistically significant (*p* > 0.05). The difference in the short-term increase of the “tumor volume” of the ablation zone is primarily due to the different ablation principles. RFA causes cell death *via* thermal coagulation necrosis, whereas MWA relies on dielectric heating. MWA’s higher power and larger active heating area produced by MW energy allow it to produce more uniform necrosis in the target lesion, making it more suitable for the treatment of larger nodules than RFA. Furthermore, in our results on disease progression, the disease progression rate in the MWA group was significantly lower than that in the RFA group, which may be related to the MWA’s higher power and energy. As a result, both MWA and RFA are considered to be treatment modalities for T1N0M0 PTC, with MWA possibly being more advantageous in the treatment of larger nodules and thorough treatment.

In terms of treatment safety, the complication rates were comparable between the two treatment modalities. The most common complications were pain and hoarseness, which all resolved spontaneously within a few days or weeks with the exception of one case in the MWA group, which had permanent hoarseness without choking. Furthermore, no life-threatening complications have been reported. As a result, we believe that MWA and RFA are safe treatments for T1N0M0 PTC.

The conducted meta-analysis still has several limitations. First, as TA is a new procedure developed in the past 15–20 years, the sample size of the studies was limited, especially in the subgroup of T1bN0M0 PTC and the LA group where we did not conduct the analysis because of insufficient data. Second, all the included studies were regionally concentrated from the same country; thus, it may produce some bias due to race. Third, most of the studies included were retrospective cohorts and all the studies were not randomized controlled trials. Fourth, the research is not compared with the current standard surgical treatment, which may make the results relatively less convincing.

## Conclusion

In conclusion, our study is currently the first meta-analysis that covers the safety and effectiveness of TA in the treatment of T1bN0M0 PTC. We discovered that the three ablation modalities are reliable and powerful for the treatment of T1aN0M0 PTC and T1bN0M0 PTC by analyzing the results of subgroup analysis. Meanwhile, MWA is better suited for the ablation of larger nodules than RFA. Considering these results, TA has a strong chance of becoming the primary treatment method for the clinical treatment of T1N0M0 PTC in the future.

## Data availability statement

The original contributions presented in the study are included in the article/supplementary material. Further inquiries can be directed to the corresponding author.

## Author contributions

M-HW and QW contributed to the conception and design of the study. M-HW performed the statistical analysis. M-HW wrote the first draft of the manuscript. XL wrote the method section of the manuscript. H-WZ revised the manuscript. All authors contributed to manuscript revision, read, and approved the submitted version.

## Funding

The work was supported by the Natural Science Foundation of Shandong Province (grant number ZR2021QH047 and ZR2021MH309) and the Clinical Science and Technology Innovation Development Program of Jinan (grant number 202134036).

## Conflict of interest

The authors declare that the research was conducted in the absence of any commercial or financial relationships that could be construed as a potential conflict of interest.

## Publisher’s note

All claims expressed in this article are solely those of the authors and do not necessarily represent those of their affiliated organizations, or those of the publisher, the editors and the reviewers. Any product that may be evaluated in this article, or claim that may be made by its manufacturer, is not guaranteed or endorsed by the publisher.
